# The Epidemiology of Invasive Group B *Streptococcus* in Denmark From 2005 to 2018

**DOI:** 10.3389/fpubh.2020.00040

**Published:** 2020-03-10

**Authors:** Hans-Christian Slotved, Steen Hoffmann

**Affiliations:** Neisseria and Streptococcus Reference Laboratory, Department of Bacteria, Parasites and Fungi, Statens Serum Institut, Copenhagen, Denmark

**Keywords:** Denmark, epidemiology, invasive, *Streptococcus agalactiae*, serotype

## Abstract

Previous epidemiology reports on invasive *Streptococcus agalactiae* (GBS) infections in Denmark did not include all patient age groups. The aim of this study was therefore to analyze the GBS incidence in all age groups during the period 2005–2018 and to present the serotype distribution and the antibiotic susceptibility. Data were retrieved from the Danish laboratory surveillance system, and these included data on typing and susceptibility testing for erythromycin and clindamycin. Early-onset disease (EOD) (mean incidence 0.17 per 1,000 live births) and late-onset disease (LOD) (mean incidence 0.14 per 1,000 live births) showed a low level during the period. The incidence was stable in the age groups 91 days to 4 years, 5–19 years, and 20–64 years. From 2005 to 2018, the incidence in the elderly showed a significantly increasing trend (*P* < 0.05), that in the 65–74 years increased from 3.23 to 8.34 per 100,000, and that in the 75+ years increased from 6.85 to 16.01 per 100,000. Erythromycin and clindamycin resistance fluctuated over the period; however, the overall trend was increasing. Data showed that EOD and LOD incidence continued to be low, whereas an increasing trend in GBS infections in the elderly was observed. The prevalence of erythromycin and clindamycin resistance increased from 2005 to 2018.

## Introduction

*Streptococcus agalactiae* [Group B *Streptococcus* (GBS)] is a commensal of the gastrointestinal tract and vagina and has been estimated to colonize the vagina in 10–35% of pregnant women (1). GBS is a well-known agent in meningitis and sepsis in newborns, and in recent years, an increasing incidence of GBS infections among the elderly, mainly bacteremia and meningitis, has been observed in the industrialized part of the world (2–4). In newborns, GBS infections are classified as early-onset disease (EOD; age 0–6 days) or late-onset disease (LOD; age 7–90 days). EOD infections are considered to be caused by vertical transmission from the mother, whereas LOD infections are mainly due to horizontal transmission from surrounding caregivers including the mother (5, 6).

Based on capsular polysaccharide antigens, 10 different serotypes of GBS have been identified, designated as Ia, Ib, and II–IX (7). The serotype distribution of colonizing GBS varies depending on the geographical region (8). A similar variation is also observed regarding invasive GBS serotypes, although five serotypes (Ia, Ib, II, III, and V) were predominant worldwide during the period 2000–2017 (9).

None of the previous studies on the epidemiology of invasive GBS infections in Denmark (6, 10–12) provided detailed information on all age groups. In these Danish studies, serotypes Ia, Ib, II, III, and V were predominant, as also observed in the global study by Madrid et al. (9). In 2001 and 2002, data from Denmark showed the presence of invasive serotype VIII isolates, which until then were observed virtually only in Japan (12). Since then, all 10 GBS serotypes have been identified in invasive infections in Denmark, although with greatly varying prevalence (13).

The aim of this study was to present the serotype distribution and the antibiotic susceptibility of submitted invasive GBS isolates from all age groups including EOD and LOD during the period 2005–2018 in Denmark.

## Materials and Methods

### Clinical Isolates

During the period 2005–2018, the national Neisseria and Streptococcus Reference (NSR) laboratory, Statens Serum Institut (SSI), received 1,875 clinical GBS isolates on the basis of voluntary submission from the departments of clinical microbiology (DCMs) from all regions of Denmark. All DCMs and nearly all hospitals in Denmark are public, and all microbiological analyses of human primary specimens are conducted at DCMs (11).

Data on invasive GBS isolates from 2005 to 2018 were retrieved from the Danish laboratory surveillance system at the NSR laboratory. Parts of the data on invasive GBS from 2005 to 2011 have already been published by Lambertsen et al. (11); however, specific data on EOD and LOD were not presented. We therefore included this period to be able to provide a complete epidemiological description of the GBS during the period from 2005 to 2018 in Denmark.

Information on age, sex, serotype, origin of the GBS isolate, and date of specimen is included in this database. All invasive GBS isolates from normally sterile sites (e.g., blood, cerebrospinal fluid, synovial fluid, pleural fluid, ascites, and tissue obtained during surgery) were included in the study like in the study by Ekelund et al. (14). Only one isolate per patient was included in this study, except if different serotypes were isolated from the same patient within 30 days or if the isolates were detected >30 days apart (11).

As described by Lambertsen et al. (10), all isolates were confirmed to be GBS by inspection of colony morphology on 5% horse blood agar plates (SSI Diagnostica, Hillerød, Denmark) and with serogrouping with group specific agglutination test using group B latex serum (Oxoid A/S, Greve, Denmark). All isolates were stored at −80°C in nutrient beef broth containing 10% glycerol (SSI Diagnostica).

### Serotyping

During 2005–2015, all isolates were serotyped using the capillary precipitation method (Lancefield method) (15), in most cases preceded by screening with GBS latex agglutination test (SSI Diagnostica, Denmark) (16). From 2016 and onwards, the serotyping procedure described by Slotved and Hoffmann (13) was used for all GBS isolates, as follows. Briefly, all isolates were serotyped using GBS latex agglutination test (SSI Diagnostica, Denmark). If the result was inconclusive, the capillary precipitation method (Lancefield method) was applied, the result of which was considered final. If this procedure did not lead to a phenotypical type designation, the isolate was categorized as being non-typable (NT) (13).

### Antibiotic Susceptibility Testing

With the use of disc diffusion, all isolates were screened for sensitivity to erythromycin (15-μg discs) and clindamycin (2-μg discs) and from 2012 also for sensitivity to penicillin G (1-μg discs). D test was performed to detect inducible clindamycin resistance. For non-susceptible isolates, the minimum inhibitory concentration (MIC) of penicillin, erythromycin, and clindamycin was determined using Etest^®^ (bioMérieux, Denmark). Antibiotic susceptibility was determined in accordance with the recommendations by EUCAST (www.eucast.org/clinical_breakpoints/).

### Data Analyses

Data were analyzed using Graph Pad Prism version 8.0.2 (GraphPad Software) for descriptive statistical analysis, calculation of Spearman *r* with confidence intervals (95% CI), and *P*-values for correlations.

For calculation of all incidence data in this manuscript, we obtained population data on both live (per 1,000) births and populations (per 100,000) for both specific age groups and total population from the Statistics Denmark homepage (https://www.dst.dk/en/Statistik, accessed 21-11-2019). RStudio version 1.1.447 and R version 3.5.0 for Windows were used for calculation of *P*-values, two-tailed Fisher's exact test, 95% CI, Cochran–Armitage test for trends, and the Kruskal–Wallis test (http://www.r-project.org/). The two-tailed Fisher's exact test was used to compare the serotype prevalence of EOD and LOD. The Kruskal–Wallis test was used to compare male vs. female incidence, and EOD vs. LOD incidence. *P* < 0.05 was considered statistically significant.

### Ethical Considerations

This was a retrospective, population-based study using national laboratory surveillance data on isolates from patients with invasive GBS infections. Because data and samples from patients were collected routinely for national surveillance purposes, no ethical approval or informed consent from patients or guardians was required. The study was approved by the Danish Data Protection Agency (record number 2007-41-0229). For further details on SSI permission to present epidemiological data, see: https://en.ssi.dk/.

## Results

During the study period, isolates from 1,875 unique cases of invasive GBS infection were received, 59 of which were from patients with meningitis (3.1%; 95% CI, 2.44–4.04).

The number of meningitis cases in each age group was 11 (EOD), 29 (LOD), 1 (91 days −4 years), 1 (5–19 years), 10 (20–64 years), 6 (65–74 years), and 1 (75+ years). Because of the very low number, no specific statistical evaluation was conducted for the meningitis group (Table 1, Supplementary Table 1).

**Table 1:** Characteristic of the invasive *Streptococcus agalactiae* (GBS) isolates received at Statens Serum Institut from 2005 to 2018.

Invasive GBS isolates from 2005 to 2018	1,875
Sex (female)	897 (47.8%)
**ISOLATE INFORMATION**	
Blood	1,732 (92.4%)
Cerebrospinal fluid	59 (3.1%)
Other sterile sites	84 (4.5%)
**AGE (YEARS) ALL PATIENTS WITH GBS INFECTION**	
Median	66.0
Age interquartile range	42.1–77.5
Age range	0–100.7


### Early-Onset Disease and Late-Onset Disease Cases

**Figure 1:**
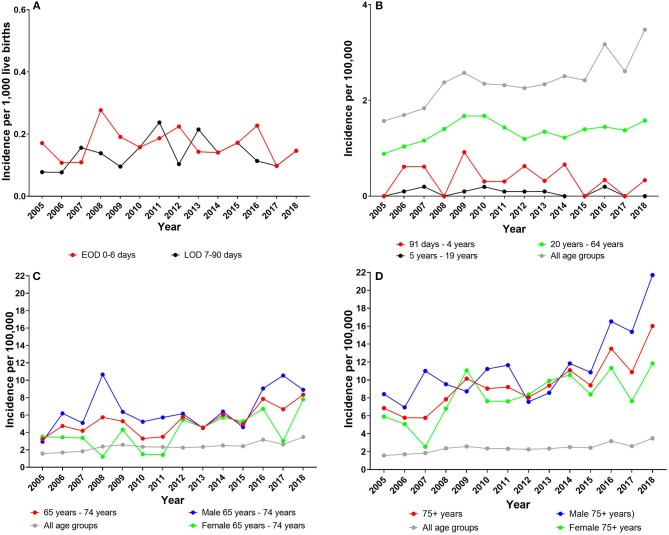
**(A)** Early-onset disease (EOD) and late-onset disease (LOD) incidence (per 1,000 live births). Incidence of all other age groups (per 100,000): **(B)** age group 91 days −64 years); **(C)** age group 65–75 years; and **(D)** age group 75+ years. **(C,D)** Also female and male incidences (per 100,000) for each age group are presented. Note the different *Y*-axes.

The incidences (per 1,000 live births) of EOD and LOD have been at a steady level since 2005 (Figure 1). The total mean incidence for EOD was 0.17 (95% CI, 0.14–0.19) and for LOD 0.14 (95% CI, 0.11–0.16). There was no significant difference between the mean incidence for EOD vs. LOD (*P* = 0.09) for the total period 2005 to 2018.

The EOD and LOD incidences did not show any significantly decreasing or increasing trend (*P* > 0.05) during the period 2005–2018, except for male LOD, which showed a significantly increasing trend (*P* = 0.04) (Table 2). There were no significant differences regarding male and female mean incidences, neither in the EOD group, nor in the LOD group (Table 2).

**Table 2:** The mean incidence rates and 95% confidence intervals for invasive *Streptococcus agalactiae* (GBS) cases, all age groups, 2005–2018.

**Age group**	**Gender**	**Mean incidence^a^ 95% CI (lower CI; upper CI)**	**Asymptotic Cochran–Armitage trend test^b^ for 2005–2018**	**Female vs. male 2005–2018 (Kruskal–Wallis test)^c^**
EOD (0–6 days)	Female	0.17 (0.12–0.21)	*P* = 0.53 (0.08)	*P* = 0.68
	Male	0.17 (0.14–0.20)	*P* = 0.59 (0.22)	
	Total	0.17 (0.14–0.19)	*P* = 0.58 (0.21)	
LOD (7–90 days)	Female	0.13 (0.10–0.17)	*P* = 0.71 (0.57)	*P* = 0.93
	Male	0.14 (0.10–0.18)	*P* = 0.04 (−1.77)^*^	
	Total	0.14 (0.11–0.16)	*P* = 0.19 (−0.89)	
91 days −4 years	Female	0.28 (0–0.56)	*P* = 0.46 (−0.09)	*P* = 0.52
	Male	0.44 (0.09–0.79)	*P* = 0.78 (0.76)	
	Total	0.36 (0.15–0.57)	*P* = 0.71 (0.54)	
5–19 years	Female	0.10 (0.01–0.19)	*P* = 0.45 (−0.13)	*P* = 0.35
	Male	0.05 (0.00–0.11)	*P* = 0.92 (1.37)	
	Total	0.08 (0.03–0.13)	*P* = 0.76 (0.72)	
20–64 years	Female	1.34 (1.15–1.53)	*P* = 0.02 (−1.97)^*^	*P* = 0.89
	Male	1.35 (1.24–1.46)	*P* = 0.22 (−0.77)	
	Total	1.35 (1.24–1.45)	*P* = 0.03 (−1.93)^*^	
65–74 years	Female	4.01 (2.95–5.24)	*P* < 0.001 (−3.65)^*^	*P* = 0.01^*^
	Male	6.60 (5.25–7.94)	*P* = 0.006 (−2.52)^*^	
	Total	5.35 (4.39–6.31)	*P* < 0.001 (−4.30)^*^	
75+ years	Female	8.19 (6.67–9.70)	*P* < 0.001 (−3.63)^*^	*P* = 0.03^*^
	Male	11.42 (9.09–13.75)	*P* < 0.001 (−4.31)^*^	
	Total	9.80 (8.36–11.25)	*P* < 0.001 (−5.73)^*^	
Total all age groups	Female	2.27 (1.95–2.59)	*P* < 0.001 (−5.10)^*^	*P* = 0.29
	Male	2.52 (2.18–2.85)	*P* < 0.001 (−5.46)^*^	
	Total	2.39 (2.17–2.61)	*P* < 0.001 (−7.48)^*^	

*EOD, early-onset disease; LOD, late-onset disease*.

a *Mean incidence: for EOD and LOD, number of cases per 1,000 live births; for all other patient categories, number of cases per 100,000 population*.

b *Trend based on Cochran–Armitage trend test*.

c *One-sided P-value*.

* *Value is statistically significant if P < 0.05*.

There was a significantly higher percentage (*P* < 0.001) of serotype III among patients with LOD (73.1%) than among patients with EOD (47.4%); and serotypes IV and VIII were only observed in the EOD group. The prevalence of the remaining serotypes did not differ significantly between EOD and LOD (Supplementary Table 2).

### Invasive *Streptococcus agalactiae* Isolates in Patients Older Than 90 Days

In the age group 91 days −4 years, the incidence (per 100,000) was 0.36 (95% CI, 0.15–0.57); in the age group 5–19 years, the incidence (per 100,000) was 0.08 (95% CI, 0.03–0.13); and in the age group 20–64 years, it (per 100,000) was 1.35 (95% CI, 1.24–1.45). In all three age groups, there was no significant difference between genders; and the incidence for the groups 91 days −4 years and 5–19 years did not show any significant change of trend (*P* > 0.05) during the period 2005–2018 (Table 2). The age group 20–64 years showed a small but significantly increasing trend (*P* = 0.03), particularly driven by the significantly increase in the female group (Table 2, Figure 1).

In the age group 65–74 years, the incidence (per 100,000) was 5.35 (95% CI, 4.39–6.31), showing a significantly increasing trend (*P* < 0.001). Regarding gender, there was a significantly higher incidence among males than among females (*P* = 0.01), although both sexes showed a significantly increasing trend (*P* < 0.05) (Table 2).

In the age group 75+ years, the incidence (per 100,000) was 9.80 (95% CI, 8.36–11.25), showing a significantly increasing trend (*P* < 0.001). There was a significantly higher incidence among males than among females (*P* = 0.03), and both sexes showed a significantly increasing trend (*P* < 0.001) (Table 2).

In the total patient population of all age groups, the combined incidence (per 100,000) was 2.39 (95% CI, 2.17–2.61), with no significant difference (*P* = 0.29) between genders. The incidence showed a significantly increasing trend (*P* < 0.001) from 1.57 per 100,000 to 3.48 per 100,000 for all age groups combined and for each gender during the period 2005 to 2018 (Figure 1, Table 2).

### Serotype Distribution

**Figure 2:**
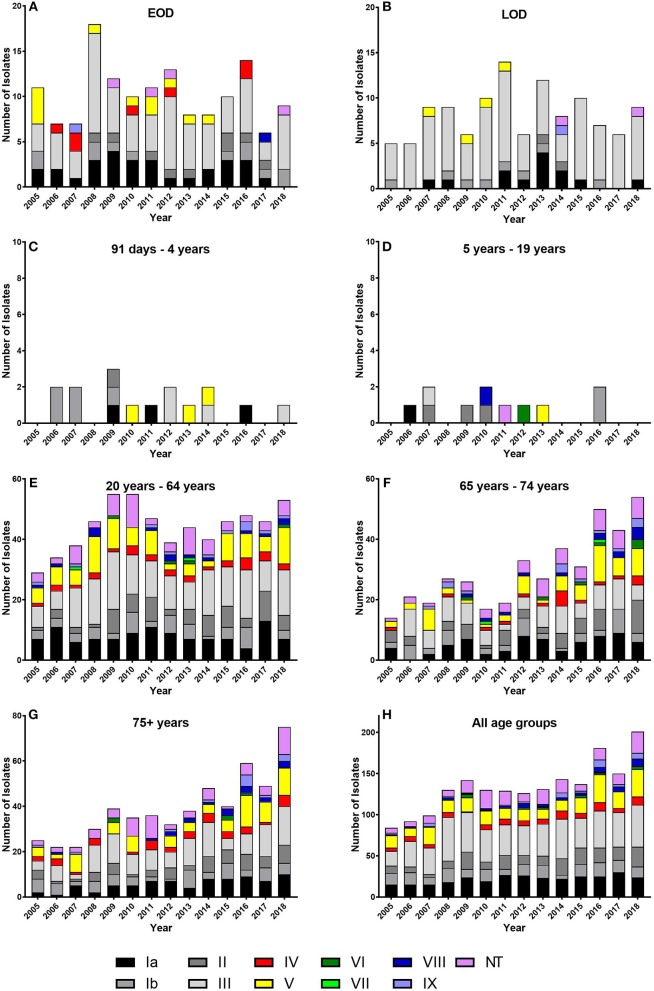
*Streptococcus agalactiae* (GBS) serotype distribution for all age groups for the period 2005–2018. **(A)** EOD, early-onset disease. **(B)** LOD, late-onset disease. **(C)** 91 days −4 years. **(D)** 5–19 years. **(E)** 20–64 years. **(F)** 65–74 years. **(G)** 75+ years. **(H)** All age groups. Note the different *Y*-axes.

All known serotypes were detected at some time point (Table 3, Figure 2). The predominant serotype was serotype III with an overall prevalence of 29.6% followed by serotypes Ia (16.4%), V (13.5%), II (10.5%), and Ib (9.7%) (Table 3). In general, serotype III was the predominant serotype in all age groups, whereas serotype V was mainly observed among the elderly (Figure 2).

**Table 3:** Serotype distribution (total number of isolates received) and resistance to erythromycin (Ery) and clindamycin (Cli) among invasive *Streptococcus agalactiae* (GBS) isolates received at Statens Serum Institut (SSI), 2005–2018.

**Serotype**	**Number of isolates**	**2005**	**2006**	**2007**	**2008**	**2009**	**2010**	**2011**	**2012**	**2013**	**2014**	**2015**	**2016**	**2017**	**2018**	**Total**	**%^a^^,^^b^**	***P*-value (Z score)^c^/*r* (95% CI) and *P*-value^d^**
Ia	Total	15	15	15	18	24	19	27	26	23	22	25	25	30	24	308	16.4	*P* = 0.69 (0.49)^c^
	Ery	1	2	1	1	4	4	7	3	4	5	10	6	5	6	59	19.2	*r* = 0.74 (0.32–0.91) *P* = 0.004^d^^*^
	Cli	0	1	1	0	1	0	1	1	1	0	2	0	2	4	14	4.5	*r* = 0.47 (−0.10–0.81) *P* = 0.09^d^
Ib	Total	14	15	9	17	10	15	7	14	16	5	15	17	15	13	182	9.7	*P* = 0.99 (2.52)^c^
	Ery	1	0	1	2	0	0	0	2	2	0	1	1	1	2	13	7.1	*r* = 0.42 (−0.16–0.78) *P* = 0.13^d^
	Cli	1	0	1	1	0	0	0	2	1	0	1	1	1	2	11	6.0	*r* = 0.26 (−0.33–0.70) *P* = 0.37^d^
II	Total	9	7	4	9	21	9	17	11	11	20	20	18	16	24	196	10.5	*P* = 0.04 (−1.74)^c^^*^
	Ery	0	1	0	2	4	4	2	2	2	6	5	4	2	6	40	20.4	*r* = 0.83 (0.52–0.95) *P* = 0.0004^d^^*^
	Cli	0	1	0	2	5	4	2	3	1	6	5	4	1	6	40	20.4	*r* = 0.81 (0.48–0.94) *P* = 0.0007^d^^*^
III	Total	18	31	32	53	48	39	37	36	39	48	36	45	42	51	555	29.6	*P* = 0.98 (2.08)^c^
	Ery	3	2	1	3	5	8	7	4	11	11	8	15	9	11	98	17.7	*r* = 0.54 (−0.01–0.84) *P* = 0.0487^d^
	Cli	3	1	2	5	7	7	7	3	10	8	8	15	9	11	96	17.3	*r* = 0.60 (0.08–0.86) *P* = 0.0261^d^^*^
IV	Total	4	6	4	6	1	6	7	6	6	10	6	10	5	10	87	4.6	*P* = 0.35 (−0.39)^c^
	Ery	2	0	0	0	0	1	0	0	0	3	0	0	0	3	9	10.3	*r* = 0.32 (−0.27–0.74) *P* = 0.27^d^
	Cli	2	0	0	0	0	1	0	0	0	3	0	0	0	2	8	9.2	*r* = 0.29 (−0.30–0.72) *P* = 0.31^d^
V	Total	15	10	21	15	17	17	13	14	12	13	19	34	20	33	253	13.5	*P* = 0.31 (−0.48)^c^
	Ery	1	2	3	10	9	6	6	5	1	2	2	6	6	5	64	25.3	*r* = 0.36 (−0.22–0.76) *P* = 0.20^d^
	Cli	1	2	3	8	9	6	6	5	2	1	2	5	6	4	60	23.7	*r* = 0.27 (−0.32–0.71) *P* = 0.35^d^
VI	Total	0	0	0	0	4	0	0	2	2	0	3	2	0	4	17	0.9	*P* = 0.055 (−1.60)^c^
	Ery	0	0	0	0	0	0	0	0	0	0	0	0	0	0	0	0	Not applicable
	Cli	0	0	0	0	0	0	0	0	0	0	0	0	0	0	0	0	Not applicable
VII	Total	0	0	1	0	0	1	0	0	1	0	0	1	0	0	4	0.2	*P* = 0.65 (0.40)^c^
	Ery	0	0	0	0	0	0	0	0	0	0	0	0	0	0	0	0	Not applicable
	Cli	0	0	0	0	0	0	0	0	0	0	0	0	0	0	0	0	Not applicable
VIII	Total	1	2	0	3	1	2	3	5	3	3	3	6	6	9	47	2.5	*P* = 0.004 (−2.66)^c^^*^
	Ery	0	1	0	0	0	0	0	0	0	0	0	0	0	0	1	2.1	*r* = −0.21 (−0.68–0.37) *P* = 0.64^d^
	Cli	0	1	0	0	0	0	0	0	0	0	0	0	0	0	1	2.1	*r* = −0.21 (−0.68–0.37) *P* = 0.64^d^
IX	Total	2	0	4	2	1	0	1	2	0	6	2	9	3	7	39	2.1	*P* = 0.01 (−2.19)^c^^*^
	Ery	0	0	0	0	0	0	0	0	0	0	0	0	0	0	0	0	Not applicable
	Cli	0	0	0	0	0	0	0	0	0	0	0	0	0	0	0	0	Not applicable
NT	Total	6	6	9	7	15	22	17	10	18	16	8	14	13	26	187	9.8	*P* = 0.22 (−0.76)^c^
	Ery	1	0	2	2	9	8	4	1	3	4	3	2	1	5	45	24.1	*r* = 0.85 (0.57–0.95) *P* = 0.0002^d^^*^
	Cli	1	0	1	2	7	6	5	1	3	1	3	2	2	2	36	19.3	*r* = 0.82 (0.50–0.94) *P* = 0.0006^d^^*^
Total	Total	84	92	99	130	142	130	129	126	131	143	137	181	150	201	1875	100.0	
	Ery	9	8	8	20	31	31	26	17	23	31	29	34	24	38	329	17.5	*P* = 0.011 (−2.29)^c^^*^
	Cli	8	6	8	18	29	24	21	15	18	19	21	27	21	31	266	14.2	*P* = 0.09 (−1.36)^c^

*The clindamycin data include the inducible clindamycin resistance isolates*.

a *The total % represents percentage of a specific serotype vs. total number of isolates*.

b *The % of resistant (Ery, Cli) serotype specific isolates vs. total number of serotype specific isolates*.

c *Asymptotic Cochran–Armitage trend test P-value (Z-score)*.

d *Spearman r correlation with 95% CI (lower CI; upper CI) and P-value, measuring the correlation between the total number of isolates and the resistance isolates*.

* *Value is statistically significant if P < 0.05*.

For the EOD group, the most prevalent serotype was serotype III (48.6%) followed by serotype Ia (20.2%) and serotype Ib (8.3%). A similar distribution was observed for the LOD group with a prevalence for serotype III at 74.1%, followed by serotype Ia (11.2%) and serotype Ib (6.9%). For the age groups 91 days −4 years and 5–19 years, the prevalence of individual serotypes were very low, with only 16 cases in total in the former age group and only 11 cases in the latter age group (Figure 2).

For the age group 20–64 years, the most prevalent serotype was serotype III (28.1%) followed by serotype Ia (18.1%), serotype V (15.2%), and serotype II (11.0%). For the age group 65–74 years the most prevalent serotype was serotype III (20.3%) followed by serotype Ia (16.7%), serotype V (14.6%), and serotype II (12.9%). The most prevalent serotypes for the age group 75+ years were serotype III (24.5%) followed by serotype Ia (14.5%), serotype V (14.4%), and serotype II (10.7%). Among the age groups EOD, LOD, and 91 days −4 years, serotypes VI and VII were not observed at all, and serotypes VIII and IX were rarely observed. Among patients 5 years or older, all serotypes were observed, although with different frequencies (Figure 2).

The prevalence of serotypes Ia, Ib, III, IV, V, VI, VII, and NT did not show any significantly increasing or decreasing trend from 2005 to 2018, whereas serotype II, VIII, and IX showed a significantly (*P* < 0.05) increasing trend for the period (Table 3).

Supplementary Table 2 shows the serotype distribution among isolates from patients with EOD and LOD.

### Demographic Development of Invasive *Streptococcus agalactiae* Cases

Comparing the different age groups and the number of received isolates, we found a stable level of GBS infection in the younger age groups, whereas there was an increasing incidence of GBS infections with increasing age (Table 2). The levels in GBS infection from 2005 to 2018 were stable for the EOD and LOD with a mean incidence of 0.17 per 1,000 (EOD) and 0.14 per 1,000 (LOD). This was also found for the age group 91 days −4 years (mean incidence 0.36 per 100,000) and the age group 5–19 years (mean incidence 0.08 per 100,000).

Regarding the three oldest age groups, we found a significantly increasing trend over the study period: the age group 20–64 years (*P* = 0.03) and the age groups 65–74 and 75+ years (*P* < 0.001).

Supplementary Figure 1 shows the percentage of the age groups 65–74 and 75+ years in the total population and among patients with GBS infection.

### Antimicrobial Susceptibility Testing

#### Penicillin

All isolates from 2012 to 2018 (in total 1069, Table 3) were tested and found to be sensitive to penicillin G.

#### Erythromycin

**Figure 3:**
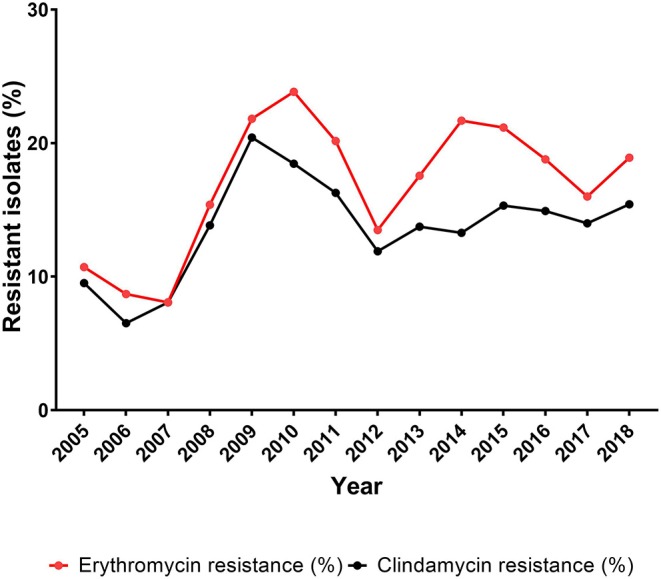
Percentage of erythromycin and clindamycin resistant isolates among all *Streptococcus agalactiae* (GBS) isolates, 2005–2018.

The erythromycin resistance rate fluctuated from 8.1% (8/99 isolates) in 2007 to 23.8% (31/130 isolates) in 2010 (Figure 3, Table 3). The percentage of erythromycin resistant GBS isolates showed a significantly increasing trend (*P* = 0.011) for the period 2005–2018 (Table 3).

#### Clindamycin

The clindamycin data include isolates with inducible clindamycin resistance. Clindamycin resistance fluctuated between 6.5% (6/92 isolates) in 2006 and 20.4% (29/142 isolates) in 2009. The percentage of clindamycin resistance showed a non-significantly increasing trend (*P* = 0.09) for the period 2005–2018 (Table 3).

#### Prevalence of the Non-susceptible *Streptococcus agalactiae* Isolates

The prevalence of non-susceptible isolates in each of the age groups below 20 years of age was very low, with a yearly number of non-susceptible isolates in the range of 1–4 cases. The majority of the non-susceptible isolates was found in the age groups 20 years or older, with a range of 2–12 cases for the 20–64 years of age, a range of 0–12 cases for the 65–74 years of age, and a range of 2–17 cases for the 75+ years of age. The significantly increasing trend (*P* = 0.013) for erythromycin resistance and non-significantly increasing trend for clindamycin resistance (Table 3) are therefore related to the increasing number of isolates observed among the patients 20 years and older (Table 2, Figure 3). This was also observed when calculating the correlation between age-related GBS infection and the prevalence of erythromycin/clindamycin non-susceptible isolates, in that a significant (*P* < 0.05) correlation was observed for the age group 20–64 years [*r* = 0.81 (95% CI, 0.47–0.94)/*r* = 0.78 (95% CI, 0.41–0.93)], the age group 65–74 years [*r* = 0.92 (95% CI, 0.76–0.98)/*r* = 0.85 (95% CI, 0.57–0.95)], and the age group 75+ years [*r* = 0.88 (95% CI, 0.65–0.96)/*r* = 0.68 (95% CI, 0.22–0.89)]. The age groups below 20 years of age did not show any significant correlation between age and the prevalence of erythromycin or clindamycin resistance (Supplementary Table 3 and Supplementary Figure 2).

Supplementary Table 3 shows the distribution of the non-susceptible GBS isolates among all age groups.

## Discussion

*Streptococcus agalactiae* is a great problem for newborns, causing EOD and LOD (2, 3). In general, two different programs for reducing the incidence of EOD have been introduced for pregnant women, the risk-based approach and the culture-based screening approach (1). Whereas, the culture-based screening has been used in the USA, the risk-based approach has been used throughout the period of this study in Denmark and is still in use (1, 6). After the introduction of programs for GBS prevention in pregnant women in some countries, a pronounced reduction has been observed, in particular regarding EOD (2, 17). However, the incidence of EOD is still high in developing countries (18).

During 1984–2002, the EOD incidence (per 1,000 live births) in Denmark ranged from 0.1 to 0.6 and the LOD incidence (per 1,000 live births) ranged from 0.0 to 0.2 (6). Ballard et al. presented data from parts of Denmark for the period 2000–2010, showing an EOD incidence of 0.18 (both Copenhagen City, Copenhagen County, and Northern Denmark) per 1,000 live births and an LOD incidence at 0.07 (Copenhagen City, Copenhagen County) and at 0.18 (Northern Denmark) per 1,000 live births (2). In the present study, the EOD and LOD incidences were nearly identical and without significant changes over the years (Figure 1, Table 2). Similar EOD and LOD incidences have also been observed in other developed countries, such as Australia, Sweden, Finland, United Kingdom, Canada, and the USA (2, 19–22). From 2005 to 2011 in Denmark, the total GBS incidence per 100,000 was around 2.0 (11). Incidence data from parts of Denmark (Copenhagen City, Copenhagen County, and Northern Denmark) in the period from 2000 to 2010 were in the range of 1.9–2.4 per 100,000 (2). In the present study, we found a similar total incidence from 2005 to 2018 (2.39 per 100,000), whereas there was a significantly increasing trend from to 1.57 per 100,000 in 2005 to 3.48 in 2018 (Figure 2, Table 2). The total incidence found in this study is comparable with the incidence detected in other developed countries, such as Iceland, Canada, Sweden, Finland, and the United Kingdom (2, 3, 19). In addition, the increase in the total GBS incidence and particular among the elderly observed in this study is in line with findings in Iceland, Finland, Norway, England and Wales, Canada, and other countries (2, 3, 19, 21, 22).

The observed increase in total GBS incidence in different countries has generally been explained by an increase of GBS infections in the elderly (2, 3, 19, 23). It has been suggested that the increase among the elderly in the developed countries can be due to an aging population with increasing prevalence of comorbidities (2, 22). This seems also to be the situation in Denmark where an increase in the general population of both age groups 65–74 and 75+ years is observed together with an increased percentage of GBS infected population of the same age groups (Supplementary Figure 1).

We found a significantly higher incidence among males 65–74 years old and 75+ years than among females in the same age groups (Table 2, Figure 1). This difference by gender was also observed by Ballard et al. (2); however, it was not observed in the study by Lamagni et al. (22). An explanation for this difference in gender-related GBS infections in Denmark cannot be established with the available data.

The serotype distribution in this study is very similar to the serotype distribution observed in other studies from developed countries (19, 21), in which it was also observed that serotypes III, Ia, and Ib were the most common serotypes in the age groups below 5 years of age; serotypes VI, VII, VIII, and IX were very rare among the age groups EOD, LOD, and 91 days −4 years; and for patients 5 years and older, all serotypes were observed, although with different frequencies. In this study, the most common serotypes in patients aged 20 years and above were serotypes III, Ia, and V (Figure 2). These serotypes were also the most prevalent serotypes among patients aged 65 years and above, which were also observed in Norway and Canada (19, 21), although they both found that serotype V was more common than serotype III. In the USA and Canada, an emergence of serotype IV has been observed in recent years in all age groups, and in some regions, serotype IV has been the second most common cause of invasive GBS in adults (20, 24). In Denmark, serotype IV was observed each year from 2005 to 2018; however, it was not observed as a predominant serotype in any of the age groups, and it constituted only 4.6% of the isolates (*n* = 87) detected in 2005–2018. Furthermore, we did not observe any significantly increasing trend for serotype IV in the period (Table 3, Figure 2). Serotype VIII was observed for the first time in Denmark in 2001 (12) and constituted 2.5% of the isolates detected from 2005 to 2018 with a significantly increasing trend (*P* < 0.05) (Table 3). This serotype was rather rare among patients younger than 20 years (Figure 2). Serotype VIII seems to be more common in Denmark compared with many other Western countries, and the prevalence resembles more the findings in Taiwan (25). This serotype is very seldomly observed in other European countries and Canada (19, 21, 26). We have no explanation for the higher prevalence in Denmark. Epidemiological studies generally find that GBS NT isolates comprise 5–10% of the detected invasive GBS isolates (3, 19, 22). In this study, the numbers of GBS NT isolates constituted 9.8% of the total number of isolates (Table 3).

The target group for GBS vaccination is generally considered to be pregnant women, although other groups, such as the elderly in general can be considered (18, 27). Currently, several capsular polysaccharide-based GBS vaccines are under development (18, 27). One of these vaccines [the hexavalent capsular polysaccharide conjugate vaccine (GBS6)] covers serotypes Ia, Ib, II, III, IV, and V (27). An evaluation of the predicted vaccine coverage for all Danish invasive GBS isolates for 2018 shows that the vaccine will cover around 77% (Table 3). The coverage for both EOD and LOD isolates will be around 89%. Although a hexavalent GBS vaccine thus theoretically could reduce EOD and LOD dramatically and reduce the overall GBS incidence by 77%, there are many reasons to assume that this might not happen in reality. For example, the GBS6 vaccine is only developed with focus on pregnant woman and not on all age groups (27). As the GBS6 vaccine covers six out of 10 known GBS serotypes, four serotypes are not included in the vaccine. As observed in this study (Table 3), there has been an increase in serotype VIII in recent years in Denmark. Furthermore, as previously mentioned, generally 5–10% of GBS isolates are NT [Table 3; (3, 19, 22)], which potentially could include unique unknown GBS serotypes. With the potential risk of non-vaccinated GBS carriers, increase in GBS serotypes not included in the GBS vaccine, and identification of possible new GBS serotypes, there is a great risk that the GBS vaccine efficacy will not meet the theoretical estimate. Although the serotype coverage of GBS vaccines and pneumococcal vaccines is very different, there is a possibility of observing serotype replacement in the GBS distribution as observed after introduction of pneumococcal vaccines (28).

Erythromycin and clindamycin are in general use in many countries, and resistance to these antibiotics is therefore monitored closely (19, 26). For erythromycin resistance, we observed an overall significantly increasing trend during the period 2005–2018, although with large fluctuations, whereas clindamycin resistance showed a non-significantly increasing trend, also with large fluctuations (Table 2, Figure 3). Moreover, there was a sharp increase in resistance rate of both antibiotics until 2009–2010, followed by a sharp decrease until 2012. These changes are very similar to changes in 2010 and 2011, respectively, reported by Martins et al. (26) and Alhhazmi et al. (19). We have not been able to identify any cause for these changes in Denmark, and the studies by Martins et al. (26) and Alhhazmi et al. (19) do not present an explanation to this observation either. During the period 2014–2017, a decrease in the prevalence of erythromycin resistance was observed, and clindamycin resistance seems to have reached a steady level (Figure 3). This development is also similar to what has been reported from Portugal (26).

The increasing prevalence of resistance toward erythromycin and clindamycin during 2012–2015 (Figure 3) is to some extent coinciding with an increasing proportion of the GBS patients being 75 years or older (Supplementary Figure 1). The developed countries are seeing an aging population with increasing burden of comorbidities requiring greater use of antibiotics (Supplementary Figure 1, Supplementary Table 2) (2, 22), thus possibly promoting development of resistance.

A weakness of this study is that it is based on voluntary submission of invasive GBS from DCMs. Previous studies in Denmark have estimated that the voluntary system results in the submission of around 58% of all invasive GBS isolates (11, 14). In the study by Ekelund et al. (14), they contacted 10 of 15 clinical microbiological departments and obtained data on their GBS positive cultures. Based on their data, it was estimated that we received 58% of all invasive isolates. The data presented in this study may therefore underestimate the incidence of GBS infections in Denmark, although a study by Ballard et al. (2) from parts of Denmark found similar GBS incidence data. Ballard et al. (2) studied invasive GBS infections in Copenhagen City, Copenhagen County, and Northern Denmark in the period from 2000 to 2010 and found an EOD incidence of 0.18 per 1,000 live births and an LOD incidence at 0.07–0.18 per 1,000 live births. In the same study, the total incidence was in the range from 1.9 to 2.4 per 100,000. These numbers correspond to the incidence found in the present study where 0.17 cases of EOD per 1,000 live births and 0.14 cases of LOD per 1,000 live births were found. The total incidence in our study during 2005–2018 was in the range 1.5–3.5 per 100,000. Also, the observed increases in GBS incidence in the elderly and some of the fluctuations observed for both antibiotic susceptibility and serotype distributions cannot be due to a sudden increase in submission of GBS isolates. We therefore believe that our data provide a representative picture of the GBS incidence in Denmark. Another limitation of the study is that we do not have data on penicillin susceptibility before 2012. However, because we did not find any penicillin non-susceptible isolates after 2012, we do not consider the absence of penicillin susceptibility data for 2005–2011 as a problem for providing a status of GBS penicillin susceptibility in Denmark.

A strength of this study is that it is nationwide and that all hospital departments are represented.

In conclusion, this study presents the GBS incidence for all age groups within the last 12 years in Denmark. We found that although EOD and LOD GBS incidences continue to be very low, we have seen high incidences showing a significantly increasing trend in GBS infections in the elderly, as also observed in other countries. Finally, we have observed an increase in serotype VIII disease in Denmark in recent years.

## Data Availability Statement

All datasets generated for this study are included in the article/Supplementary Material.

## Author Contributions

H-CS designed the study, analyzed the data, and drafted the manuscript. SH analyzed and reviewed the data, contributed to the manuscript, and critically revised the manuscript. Both authors have approved the final manuscript.

### Conflict of Interest

The authors declare that the research was conducted in the absence of any commercial or financial relationships that could be construed as a potential conflict of interest.
