# Supplementary material for: The Epidemiology of Invasive Group B Streptococcus in Denmark From 2005 to 2018
Source: Front Public Health. 2020 Mar 10;8:40. doi: 10.3389/fpubh.2020.00040 (PMC7076979; doi:10.3389/fpubh.2020.00040)
Supplement: Supplementary file 1 [file Data_Sheet_1.docx]

Supplementary tables and figures.

**Article Title: T**he Epidemiology Of Invasive Group B *Streptococcus* In Denmark From 2005 To 2018

**Authors:** Hans-Christian Slotved^1^*, Steen Hoffmann^1^.

**Affiliations:**

1. Neisseria and Streptococcus Reference Laboratory, Department of Bacteria, Parasites and Fungi, Statens Serum Institut, Copenhagen, Denmark.

***Corresponding author:** Hans-Christian Slotved, Department of Bacteria, Parasites and Fungi,

Bldg. 47/119, Artillerivej 5, DK-2300 Copenhagen S, Denmark.

Phone: +45 32688422, E-mail: [hcs@ssi.dk](mailto:hcs@ssi.dk)

Supplementary table 1. Data on meningitis cases per year per age group.

| Year | Sepsis | Other* | Total | Meningitis | Meningitis  (% of total) | Meningitis cases per age group | | | | | | |
| --- | --- | --- | --- | --- | --- | --- | --- | --- | --- | --- | --- | --- |
|  |  |  |  |  |  | EOD^a^ | LOD^b^ | Total  (91d - 4y) | Total  (5y -19y) | Total  (20y -64y) | Total  (65y -74y) | Total  (75y+) |
| 2005 | 77 | 4 | 85 | 4 | 4.7 | 0 | 1 | 0 | 0 | 3 | 0 | 0 |
| 2006 | 83 | 5 | 92 | 4 | 4.3 | 1 | 2 | 0 | 0 | 0 | 0 | 1 |
| 2007 | 93 | 3 | 100 | 4 | 4.0 | 0 | 2 | 1 | 0 | 1 | 0 | 0 |
| 2008 | 116 | 8 | 130 | 6 | 4.6 | 4 | 2 | 0 | 0 | 0 | 0 | 0 |
| 2009 | 119 | 18 | 142 | 5 | 3.5 | 1 | 2 | 0 | 0 | 1 | 1 | 0 |
| 2010 | 109 | 21 | 130 | 0 | 0 | 0 | 0 | 0 | 0 | 0 | 0 | 0 |
| 2011 | 117 | 5 | 129 | 7 | 5.4 | 2 | 4 | 0 | 0 | 1 | 0 | 0 |
| 2012 | 121 | 3 | 126 | 2 | 1.6 | 0 | 1 | 0 | 0 | 0 | 1 | 0 |
| 2013 | 123 | 3 | 131 | 5 | 3.8 | 0 | 3 | 0 | 1 | 0 | 1 | 0 |
| 2014 | 137 | 2 | 141 | 2 | 1.4 | 0 | 2 | 0 | 0 | 0 | 0 | 0 |
| 2015 | 127 | 3 | 137 | 7 | 5.1 | 0 | 4 | 0 | 0 | 2 | 1 | 0 |
| 2016 | 173 | 3 | 181 | 5 | 2.8 | 2 | 2 | 0 | 0 | 1 | 0 | 0 |
| 2017 | 146 | 2 | 150 | 2 | 1.3 | 0 | 1 | 0 | 0 | 0 | 1 | 0 |
| 2018 | 191 | 4 | 201 | 6 | 3.0 | 1 | 3 | 0 | 0 | 1 | 1 | 0 |
| Total | 1732 | 84 | 1875 | 59 | 3.1 | 11 | 29 | 1 | 1 | 10 | 6 | 1 |

Other*: Represent synovial fluid, pleural fluid, ascites, and tissue obtained during surgery.

^a^Incidence of early-onset disease (EOD)

^b^Late-onset disease (LOD)

Supplementary table 2. Serotype distribution of invasive GBS isolates in patients with EOD and LOD, 2005 - 2018.

| Serotype | EOD^a^ | LOD^b^ | P-values^c^ |
| --- | --- | --- | --- |
| Ia | 29 | 13 | 0.06 |
| Ib | 12 | 8 | 0.82 |
| II | 9 | 2 | 0.12 |
| III | 70 | 86 | < 0.001* |
| IV | 7 | 0 | 0.018* |
| V | 11 | 4 | 0.19 |
| VI | 0 | 0 | NA |
| VII | 0 | 0 | NA |
| VIII | 1 | 0 | 1.0 |
| IX | 1 | 1 | 1.0 |
| NT | 4 | 2 | 0.69 |
| Total | 144 | 116 |  |

^a^Early-onset disease (EOD)

^b^Late-onset disease (LOD)

^c^The P-value was calculated by two-tailed Fisher’s Exact Test.

* Significant.

NA = Not applicable; NT = non-typable

Supplementary table 3. The distribution of erythromycin and clindamycin resistant isolates according to patient age groups, 2005 - 2018.

| Year |  | EOD^a^ | LOD^b^ | Total  (91d - 4y) | Total  (5y -19y) | Total  (20y -64y) | Total  (65y -74y) | Total  (75y+) |
| --- | --- | --- | --- | --- | --- | --- | --- | --- |
| 2005 | Total | 1 | 0 | 0 | 0 | 4 | 0 | 4 |
|  | Ery | 1 | 0 | 0 | 0 | 4 | 0 | 4 |
|  | Cli | 1 | 0 | 0 | 0 | 3 | 0 | 4 |
| 2006 | Total | 1 | 0 | 0 | 0 | 2 | 0 | 2 |
|  | Ery | 1 | 0 | 0 | 0 | 2 | 0 | 2 |
|  | Cli | 1 | 0 | 0 | 0 | 2 | 0 | 2 |
| 2007 | Total | 1 | 1 | 0 | 0 | 4 | 1 | 3 |
|  | Ery | 1 | 1 | 0 | 0 | 3 | 1 | 2 |
|  | Cli | 1 | 0 | 0 | 0 | 3 | 1 | 3 |
| 2008 | Total | 2 | 1 | 0 | 0 | 6 | 2 | 4 |
|  | Ery | 1 | 1 | 0 | 0 | 6 | 2 | 3 |
|  | Cli | 2 | 1 | 0 | 0 | 6 | 0 | 3 |
| 2009 | Total | 1 | 1 | 0 | 0 | 9 | 3 | 5 |
|  | Ery | 0 | 1 | 0 | 0 | 9 | 3 | 2 |
|  | Cli | 1 | 1 | 0 | 0 | 6 | 1 | 4 |
| 2010 | Total | 3 | 0 | 0 | 0 | 9 | 0 | 3 |
|  | Ery | 3 | 0 | 0 | 0 | 9 | 0 | 2 |
|  | Cli | 2 | 0 | 0 | 0 | 7 | 0 | 2 |
| 2011 | Total | 2 | 1 | 1 | 1 | 11 | 4 | 7 |
|  | Ery | 2 | 1 | 1 | 1 | 10 | 4 | 7 |
|  | Cli | 2 | 1 | 0 | 1 | 9 | 4 | 6 |
| 2012 | Total | 1 | 3 | 0 | 0 | 3 | 5 | 5 |
|  | Ery | 1 | 3 | 0 | 0 | 3 | 5 | 4 |
|  | Cli | 1 | 3 | 0 | 0 | 2 | 5 | 3 |
| 2013 | Total | 1 | 3 | 0 | 0 | 9 | 4 | 5 |
|  | Ery | 1 | 3 | 0 | 0 | 9 | 4 | 5 |
|  | Cli | 1 | 1 | 0 | 0 | 8 | 3 | 3 |
| 2014 | Total | 2 | 4 | 0 | 0 | 6 | 7 | 12 |
|  | Ery | 2 | 4 | 0 | 0 | 6 | 7 | 12 |
|  | Cli | 1 | 2 | 0 | 0 | 3 | 6 | 7 |
| 2015 | Total | 3 | 2 | 0 | 0 | 12 | 7 | 5 |
|  | Ery | 3 | 2 | 0 | 0 | 12 | 7 | 5 |
|  | Cli | 3 | 2 | 0 | 0 | 8 | 7 | 1 |
| 2016 | Total | 1 | 2 | 0 | 0 | 9 | 12 | 10 |
|  | Ery | 1 | 2 | 0 | 0 | 9 | 12 | 10 |
|  | Cli | 1 | 2 | 0 | 0 | 8 | 9 | 7 |
| 2017 | Total | 1 | 1 | 0 | 0 | 7 | 8 | 9 |
|  | Ery | 1 | 1 | 0 | 0 | 7 | 7 | 8 |
|  | Cli | 1 | 1 | 0 | 0 | 4 | 7 | 8 |
| 2018 | Total | 2 | 3 | 0 | 0 | 8 | 11 | 17 |
|  | Ery | 2 | 3 | 0 | 0 | 8 | 11 | 15 |
|  | Cli | 1 | 3 | 0 | 0 | 6 | 9 | 14 |
| r (95% CI) and  P value^c^ | Ery | -0.21 (-0.68 – 0.37)  P = 0.46 | 0.25 (-0.34 – 0.70) P = 0.38 | - 0.04 (-0.57 – 0.52) P > 0.99 | 0.16 (-0.42 – 0.65) P = 0.47 | 0.81 (0.47 – 0.94) P = 0.0008* | 0.92 (0.76 – 0.98) P < 0.0001* | 0.88 (0.65 – 0.96) P < 0.0001* |
|  | Cli | 0.19 (-0.40 – 0.66)  P = 0.52 | 0.06 (-0.50 – 0.58) P = 0.85 | Not applicable | 0.16 (-0.42 – 0.65) P = 0.47 | 0.78 (0.41 – 0.93) P = 0.0016* | 0.85 (0.57 – 0.95) P = 0.0003* | 0.68 (0.22 – 0.89) P = 0.0085* |

^a^Incidence of early-onset disease (EOD)

^b^Late-onset disease (LOD)

^c^Spearman r correlation with 95% CI (lower CI; upper CI) and P value, measuring the correlation between the total number of isolates and the resistance isolates. * Value is statistically significant if P<0.05.

Ery = erythromycin resistance

Cli = clindamycin resistance

Supplementary figure 1. The percentage of the age groups 65 - 74y and 75y+ of the general population, and the percentage of patients in the same two age groups among all patients with GBS infection.

Supplementary figure 2. Seven age specific graphs showing the correlation between the annual number of isolates versus annual number of resistant isolates for both erythromycin and clindamycin. Each triangle represents data from one year. Note the different axes.
